# Fine-Scale Ecological and Genetic Population Structure of Two Whitefish (Coregoninae) Species in the Vicinity of Industrial Thermal Emissions

**DOI:** 10.1371/journal.pone.0146656

**Published:** 2016-01-25

**Authors:** Carly F. Graham, Rebecca L. Eberts, Thomas D. Morgan, Douglas R. Boreham, Stacey L. Lance, Richard G. Manzon, Jessica A. Martino, Sean M. Rogers, Joanna Y. Wilson, Christopher M. Somers

**Affiliations:** 1 Department of Biology, University of Regina, Regina, Saskatchewan, Canada; 2 Medical Sciences, Northern Ontario School of Medicine, Greater Sudbury, Ontario, Canada; 3 Savannah River Ecology Laboratory, University of Georgia, Athens, Georgia, United States of America; 4 Department of Biological Sciences, University of Calgary, Calgary, Alberta, Canada; 5 Department of Biology, McMaster University, Hamilton, Ontario, Canada; Bournemouth University, UNITED KINGDOM

## Abstract

Thermal pollution from industrial processes can have negative impacts on the spawning and development of cold-water fish. Point sources of thermal effluent may need to be managed to avoid affecting discrete populations. Correspondingly, we examined fine-scale ecological and genetic population structure of two whitefish species (*Coregonus clupeaformis* and *Prosopium cylindraceum*) on Lake Huron, Canada, in the immediate vicinity of thermal effluent from nuclear power generation. Niche metrics using δ^13^C and δ^15^N stable isotopes showed high levels of overlap (48.6 to 94.5%) in resource use by adult fish captured in areas affected by thermal effluent compared to nearby reference locations. Isotopic niche size, a metric of resource use diversity, was 1.3- to 2.8-fold higher than reference values in some thermally affected areas, indicative of fish mixing. Microsatellite analyses of genetic population structure (F_st_, STRUCTURE and DAPC) indicated that fish captured at all locations in the vicinity of the power plant were part of a larger population extending beyond the study area. In concert, ecological and genetic markers do not support the presence of an evolutionarily significant unit in the vicinity of the power plant. Thus, future research should focus on the potential impacts of thermal emissions on development and recruitment.

## Introduction

Worldwide a variety of energy generating stations use natural lake or river water as an industrial coolant, which may be released as warmed effluent into the environment. Thermal pollution of this nature can result in localized changes in water temperature ranging from 2 to 15°C above ambient values (e.g., [1–6]). Freshwater fish species, especially those with narrow temperature tolerances, may be negatively affected by thermal pollution (e.g., [5–8]). Previous studies have shown that thermal effluents can reduce reproduction and survival [3,7,9], cause changes in species movement and distribution [4,10,11] and alter habitat structure [6]. Individually or collectively, thermal effluent may be associated with local extirpation of sensitive fish species. Ideally, thermal effluent should be managed with population genetic considerations to avoid impacting fish that are distinct populations.

The population structure of fish is often assessed using neutral genetic markers to identify discrete subpopulations that result from reduced gene flow due to a variety of factors, including geographic barriers [12–16], human impacts [17,18] and spawning site fidelity [19–21]. The polymorphism of microsatellites makes them ideal markers to identify fine-scale population structure within many different species [15,22–26]. Genetic data provide excellent insight into long-term aspects of population structure and have been used extensively in fisheries management (e.g., [19,21,27,28]). Important management decisions can be aided using genetic markers; however, many studies often lack ecological data to corroborate genetic findings.

Stable isotopes of carbon and nitrogen are ecological markers that provide a quantitative means of comparing resource use among groups of fish. The range and variance of isotopic values present a two-dimensional metric of niche that incorporates information about carbon source, a proxy for habitat in freshwater, and nitrogen source, indicative of trophic position [29–31]. Together these isotopic values describe resource use [32–34]. Groups of fish with different isotopic niches are comprised of individuals from different food webs or employing different feeding strategies (e.g., [35]). Thus, stable isotopes may be used to identify fish population structure based on resource use [36]. This metric of structure is shorter-term than genetic markers, reflecting the metabolic turnover time of the tissues analyzed [37]. For spawning aggregations, analysis of isotopic population structure using muscle tissue reveals whether groups used similar or different resources prior to aggregating. Despite the potential advantages of stable isotopes, few fisheries studies have used this approach to assess spatial population structure of individual species (but see [35,36]).

Canada currently has four major nuclear power generating stations on the Laurentian Great Lakes, the largest freshwater system in the world. All three stations operate multiple CANDU reactors and use lake water for once-through cooling processes to cool steam condensers [38–42]. The largest power generating station is located on Douglas Point in eastern Lake Huron, where it releases large volumes of warmed water back into the lake. Of concern are potentially negative impacts of thermal emissions on the reproduction and development of members of the Coregoninae subfamily (Salmonidae family), which are fall-spawning species that require a narrow range of cold temperatures for successful embryogenesis [43–45]. Specifically, lake whitefish (*Coregonus clupeaformis*) comprise one of the most important commercial fisheries within the Great Lakes [46–49], and round whitefish (*Prosopium cylindraceum*) may be an important indicator species for environmental monitoring [50]. Recent studies have found that increases in incubation temperature resulted in earlier hatch and decreased survival in both whitefish species [51–53]. Early hatch can have potential ecological impacts (e.g. altered predation and food availability), increasing the importance of development temperature beyond morphological effects [54–56]. From a conservation and management perspective, it is important to know whether fish spawning in areas receiving thermal emissions are ecologically and genetically distinct from those in reference areas.

Both genetic and ecological population structuring occur among lake whitefish spawning aggregations in Lake Huron on the spatial scale of hundreds of kilometers. Previous studies have identified genetic differentiation among populations in different basins or separated by large geographic distances (e.g., [14,19,57–59]). Lake whitefish also vary in diet [60] and habitat use [58] in different areas of the lake, and historically have undergone resource use shifts in association with changes in the environment [61–64]. Previous studies of lake whitefish provide excellent evidence of barriers to gene flow and resource use heterogeneity within Lake Huron. However, they were not designed to address questions about fine-scale population structure, such as whether or not fish in the immediate vicinity of point source emissions are distinct from those in nearby reference areas. In addition, very little is known about any aspect of population structure for round whitefish.

In this study we examined fine-scale population structure of spawning lake and round whitefish in the region around thermal emissions from the nuclear power generating station on Douglas Point, Lake Huron. We used niche analysis based on stable isotopes and neutral genetic variation from microsatellites in concert to investigate the ecological and genetic population structure of these fish. Our focus was on fine-scale population structure to address the specific question of whether fish in areas affected by thermal emissions were distinct from adjacent reference areas. For each fish species our specific objectives were to compare the following metrics in areas affected by thermal emissions vs. adjacent reference zones: (1) isotopic niche characteristics; and (2) genetic population structure. The results of this study will inform whitefish management within the study area.

## Materials and Methods

### Study Area and Species

**Fig 1:**
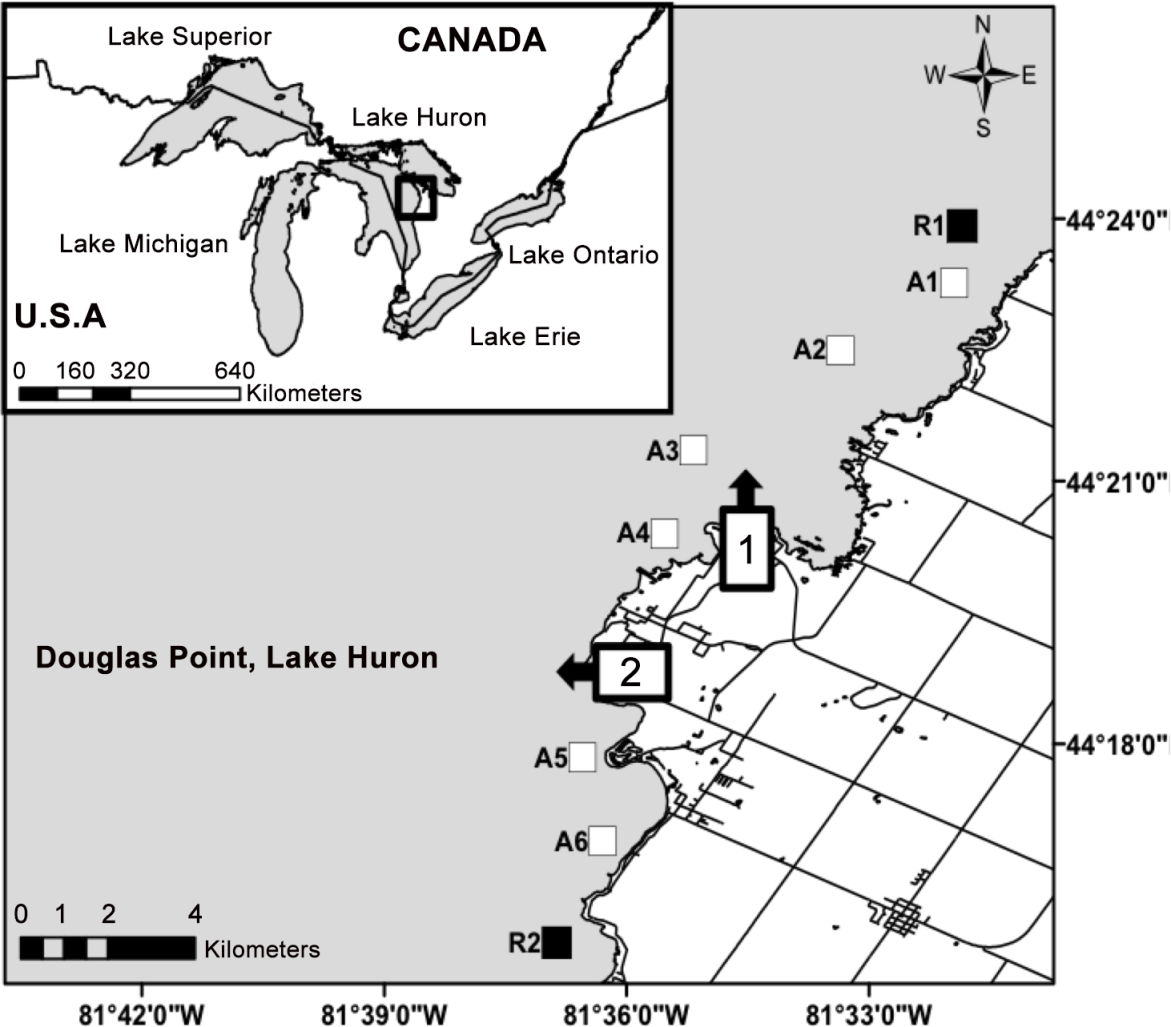
Map of the study area on eastern Lake Huron, Canada (inset) showing the location of two nuclear power generating stations (1 and 2), each operating 4 CANDU reactors. The locations of outflow channels for thermal effluent are indicated by arrows. Gill netting areas for Lake and Round Whitefish are indicated. A1-A6 are sites potentially affected by warmer temperatures (an average of 3°C [65]), whereas sites R1 and R2 are reference locations outside of the thermal plume. Fish were collected in the falls of 2010 and 2011 at each site.

Our study took place in the vicinity of the Bruce Power site in eastern Lake Huron, Ontario, Canada (Fig 1; 44°19’35.39 N, 81°36’01.22 W). Nuclear power has been produced at this site using CANDU reactor technology since 1968, and the current operation consists of 8 CANDU reactors divided into two generating stations (identified as 1 and 2 in Fig 1). Station 1, also called Bruce A, has an intake and discharge rate of 175,000 L/s, whereas station 2 (Bruce B) has an intake and discharge rate of 193,000 L/s. The maximum permitted difference between discharge and ambient water temperatures for both stations is 11.1°C from April 15 to December 14, and 13.0°C from December 15 to April 14 each year. The extent and movement of the thermal plume resulting from warm water discharge at this site is highly variable. The area potentially affected by both discharges combined is predicted to range from 70 to 3,600 ha in near-shore parts of Lake Huron adjacent to the generating stations [42]. To facilitate monitoring and environmental impact assessment, Bruce Power worked with a consulting company [65] to identify 8 sampling zones for fish in relevant near-shore habitats: 2 reference areas outside of the influence of the thermal plume (R1 and R2 in Fig 1), and 6 areas shown to experience warming from thermal discharge ([65] A1-A6 in Fig 1). These zones were found to have suitable habitat and attracted mature whitefish during spawning [65,66]. Research by Thome *et al*. [67] has shown that within the potentially affected sites the temperature is highly variable with an average winter temperature of up to 3°C warmer than ambient. We used these areas as the study design for population structure assessment. All 8 areas contain at least some rock-cobble substrate in the 2-8m depth range preferred by spawning whitefish, and adults in spawning condition have been collected throughout the study area. However, the importance of the region for whitefish spawning is currently unclear.

Both whitefish species we studied are broadcast spawners that cast their eggs over reefs and rock-cobble substrate in shallow (2-8m depth) near-shore areas in the late fall [68]. Lake whitefish spawn from October to mid December and round whitefish spawn during early November to early December [43,44,47,51,69,70]. Whitefish eggs normally incubate over winter, with optimal development temperatures of 0.5°C and 4.5°C for lake and round whitefish, respectively [71]. Laboratory studies that manipulate temperature show that mortality and deformity rates increase significantly when eggs are incubated at warmer temperatures [51–53]. Further, within Lake Huron whitefish have faced many recent challenges, including system-level changes with the invasion of dreissenid mussels [61–63,72], introduction of non-native predators [47,73] and the degradation of habitat [47].

### Sample Collection

All animal research was approved by the University of Regina President's Committee on Animal Care, following the guidelines of the Canadian Council on Animal Care. The approved Animal Use Protocol was AUP 11–13 "Population and Conservation Genetics of Freshwater Fish". The authors did not require a permit to sample fish; the permit was issued to Bruce Power and fish specimens were received after collection

Adult lake and round whitefish were collected as part of Bruce Power’s environmental assessment follow-up program (see [66]) using bottom-set gill nets from the 8 areas described above (2 reference and 6 affected sites), which span approximately 24 km of shoreline (Fig 1). Methods are described in detail elsewhere [66,67]. In brief, nets (457 m length x 1.5m height; 5.7 cm and 11.4 cm mesh size) were set overnight for 19–24 hours on 13 dates in 2010 (Oct 25 –Dec 4) and 15 dates in 2011 (Oct 27 –Dec 13) at 4.5–6 m depths in areas with cobble and boulder substrate. Fish captured in gill nets were examined for morphological measurements and confirmed to be sexually mature. Fish were retained so that we could collect a 5 x 2 x 2 cm piece of dorsal muscle, which was frozen for later DNA extraction and stable isotopes analysis. A total of 336 lake and 319 round whitefish were used for stable isotope analysis, and 208 lake and 327 round whitefish were used for the genetic analysis (Table 1).

**Table 1:** Lake and round whitefish collected via multi-panel gillnets during 2010 and 2011 spawning seasons at 8 locations in the Douglas Point area of Lake Huron. Gill net sites were chosen to reflect two reference areas (R1, R2) and 6 potentially thermally affected areas (A1-A6). Muscle tissue was collected for stable isotopes and microsatellite analyses from fish at each site.

Site	Latitude	Longitude	Stable Isotopes (n)		Genetic Analyses (n)	
			Lake	Round	Lake	Round
R1	44°23'56" N	81°31'51" W	87	53	30	65
A1	44°22'31" N	81°33'21" W	32	40	30	38
A2	44°21'22" N	81°35'10" W	66	43	30	43
A3	44°20'25" N	81°35'32" W	36	28	30	26
A4	44°17'51" N	81°36'33" W	17	22	14	21
A5	44°16'54" N	81°36'18" W	32	42	30	37
A6	44°15'44" N	81°36'52" W	15	33	15	31
R2	44°23'17" N	81°31'57" W	51	58	29	66
***Total***			**336**	**319**	**208**	**327**

### Stable Isotopes

Sub-samples of dorsal muscle from individuals from reference and affected sites were rinsed with distilled water, dehydrated in a drying oven at 50°C for 120–168 hours, ground to a fine powder using a dental amalgamator and weighed (0.5–1.0 mg) into tin capsules (Table 1). Stable isotopes values from muscle reflect incorporation for several months [74], which ensured that we compared resource use by fish prior to spawning. Weighed samples were analyzed for carbon and nitrogen stable isotopes using a Thermo Finnigan Delta V isotope ratio mass spectrometer (Institute for Environmental Change and Society, University of Regina). We ran unknowns, laboratory standards of wheat and bovine liver, and replicates of both unknowns and standards in each batch. Isotope values are reported as *δ* ^13^C_VPDB_ and *δ* ^15^N_AIR_ (delta values) in units of per mil (*‰*). Replicates of standards and unknowns generally varied less than 0.2 *‰*. We arithmetically lipid-corrected *δ* ^13^C with the McConnaughey & McRoy [75] formula when C:N ratios were greater than 3.5.

We created Bayesian standard ellipses using SIBER Metrics to define and compare the isotopic niches for each species at each sample site. Bayesian ellipses encompass 40% of the data, thus this metric enables a robust comparison of niche that is less biased by differences in sample size than traditional metrics [76,77]. The area encompassed by standard ellipses (SEA) is a measure of isotopic niche size (*‰*^2^) and is directly related to resource use diversity [76]. We corrected SEA for small sample size (SEA_*c*_; [76]). The SEA of lake and round whitefish at each site was calculated with 10,000 replications, creating a Bayesian SEA (SEA_*b*_) to obtain an average SEA with 95% credibility. To compare resource use similarity between fish in the reference and affected sites we quantified isotopic niche overlap by calculating the area shared between standard ellipses of each reference site with each affected site. To compare resource use diversity we calculated the Bayesian probability that SEA of fish in affected sites was larger than fish in reference sites based on SEA_*b*_ estimations. All niche metrics were calculated with the Stable Isotope Analysis in R (SIAR) package [78,79].

### Genetic Analysis

We extracted genomic DNA from 20 mg of dorsal muscle tissue following the manufacturer’s guidelines (Genomic DNA Isolation Kit, Norgen Bioteck Corp., Ontario, Canada), with the exception that we added 28 U of RNase A (Qiagen Inc., Ontario, Canada) and extended proteinase K digestion to 8–12 hours at 56°C. DNA was quantified using a Qubit 2.0 Fluorometer (Life Technologies Inc., Ontario, Canada) prior to diluting to a standard concentration (5 ng/μl) for PCR reactions. Lake whitefish were genotyped at 9, and round whitefish at 6 microsatellite loci previously developed for each species (lake whitefish: [17,80]; round whitefish: [81]). In addition, lake whitefish were genotyped with 11 and round whitefish with 5 additional tetranucleotide microsatellite loci that were developed specifically for this study using the methods described in Lance *et al*. ([82]; S1 Table).

Microsatellite loci were amplified in multiplexed PCR reactions using 2 sets of primer pairs with the reverse primer of each pair labeled with fluorescent dyes (WellRED D3 and D4 dyes, Integrated DNA Technologies, Ontario, Canada). PCR reactions were performed in a total reaction volume of 25 μL containing 1X PCR Master Mix (Norgen Bioteck Corp., Ontario, Canada), 2 μM forward and reverse primer, and 10 ng of template DNA. Thermocycling conditions for whitefish primers were: 5 min at 95°C, followed by 30 cycles at 95°C for 30 s, 30 s at the locus-specific annealing temperature (see Table 2), 60 s at 72°C and a final extension of 5 min at 72°C. The touchdown conditions started with 5 min denaturation at 95°C, followed by 20 cycles at 95°C for 30 s, 30 s at 65°C (decreasing by 0.5°C per cycle), 30 s at 72°C, and then 20 cycles at 95°C for 30 s, 30 s at 55°C, 30 s at 72°C followed by a final extension for 5 min at 72°C. Negative controls without DNA template were run for all samples.

**Table 2:** Details of 31 polymorphic microsatellite loci used to genotype lake and round whitefish. TD refers to a touchdown PCR approach where the annealing temperature ranged from 65°C to 55°C.

Loci	Species	Annealing Temperature (°C)	Reference
BWF1	Lake whitefish	50	Patton et al., 1997
BWF2	Lake whitefish	50	Patton et al., 1997
Cocl Lav1	Lake whitefish	55	Rogers et al., 2004
Cocl Lav4	Lake whitefish	57	Rogers et al., 2004
Cocl Lav6-di	Lake whitefish	60	Rogers et al., 2004
Cocl Lav6-tetra	Lake whitefish	TD65	This study*
Cocl Lav12	Lake whitefish	TD65	This study*
Cocl Lav18	Lake whitefish	TD65	This study*
Cocl Lav19	Lake whitefish	57	Rogers et al., 2004
Cocl Lav20	Lake whitefish	TD65	This study*
Cocl Lav27	Lake whitefish	55	Rogers et al., 2004
Cocl Lav33	Lake whitefish	TD65	This study*
Cocl Lav34	Lake whitefish	TD65	This study*
Cocl Lav43	Lake whitefish	TD65	This study*
Cocl Lav44	Lake whitefish	TD65	This study*
Cocl Lav45-di	Lake whitefish	60	Rogers et al., 2004
Cocl Lav45-tetra	Lake whitefish	TD65	This study*
Cocl Lav47	Lake whitefish	TD65	This study*
Cocl Lav48	Lake whitefish	TD65	This study*
Cocl Lav68	Lake whitefish	57	Rogers et al., 2004
Prwi6	Round whitefish	TD65	O’Bryhim et al., 2013
Prwi15	Round whitefish	TD65	O’Bryhim et al., 2013
Prwi24	Round whitefish	TD65	O’Bryhim et al., 2013
Prwi25	Round whitefish	TD65	O’Bryhim et al., 2013
Prwi27	Round whitefish	TD65	O’Bryhim et al., 2013
Prwi28	Round whitefish	TD65	O’Bryhim et al., 2013
Prwi55	Round whitefish	TD65	This study*
Prwi56	Round whitefish	TD65	This study*
Prwi60	Round whitefish	TD65	This study*
Prwi65	Round whitefish	TD65	This study*
Prwi72	Round whitefish	TD65	This study*

*More details on the loci developed for this study are presented in S1 Table.

PCR products were size fractionated using a Beckman Coulter GenomeLab GeXP Genetic Analysis System with a 400 bp internal size standard (Beckman Coulter, Mississauga, ON). Alleles were scored using GENEMARKER 2.20 software (Softgenetics, State College, PA) under default settings, with the exception of a bin width of 1 nucleotide to reflect the resolution limit of capillary electrophoresis. Automated allele calling was verified manually and confirmed by a second, independent observer. The program MICRO-CHECKER [83] was used to quality check all microsatellite profiles by estimating the potential frequency of null alleles and the observed and expected heterozygosities (H_O_ and H_E_). Deviations from Hardy-Weinberg Equilibrium (HWE) were examined for each locus using GENEPOP v4.3 [84].

Population structure among spawning groups was assessed using several different approaches. We calculated fixation indices (F_ST_; [85]) in the program GENODIVE [86]. F_ST_ values were evaluated by comparing reference and affected regions, individual sampling sites, and sampling years for both lake and round whitefish. Second, we used Bayesian clustering in the program STRUCTURE to identify the number of potential populations in our data sets [87]. The analysis was repeated 10 times for each value of K ranging from 1 to 8 to ensure consistency between runs. We used a burn-in of 100,000 steps and 100,000 MCMC steps. We also calculated the value of ΔK for the data using the methods of Evanno *et al*. [88]. Only loci that were in HWE were included in F_ST_ and Bayesian analyses. Finally, the data were analyzed using Discriminant Analysis of Principle Components (DAPC), a multivariate ordination method in the R package ADEGENET [89]. DAPC does not require the assumption of HWE, so all loci were included in this analysis. We generated ellipses using ADEGENET for each site by using 65 and 46 principal components (one third of the total) for lake and round whitefish, respectively, to avoid over-fitting the discriminant functions.

## Results

### Stable Isotopes

**Fig 2:**
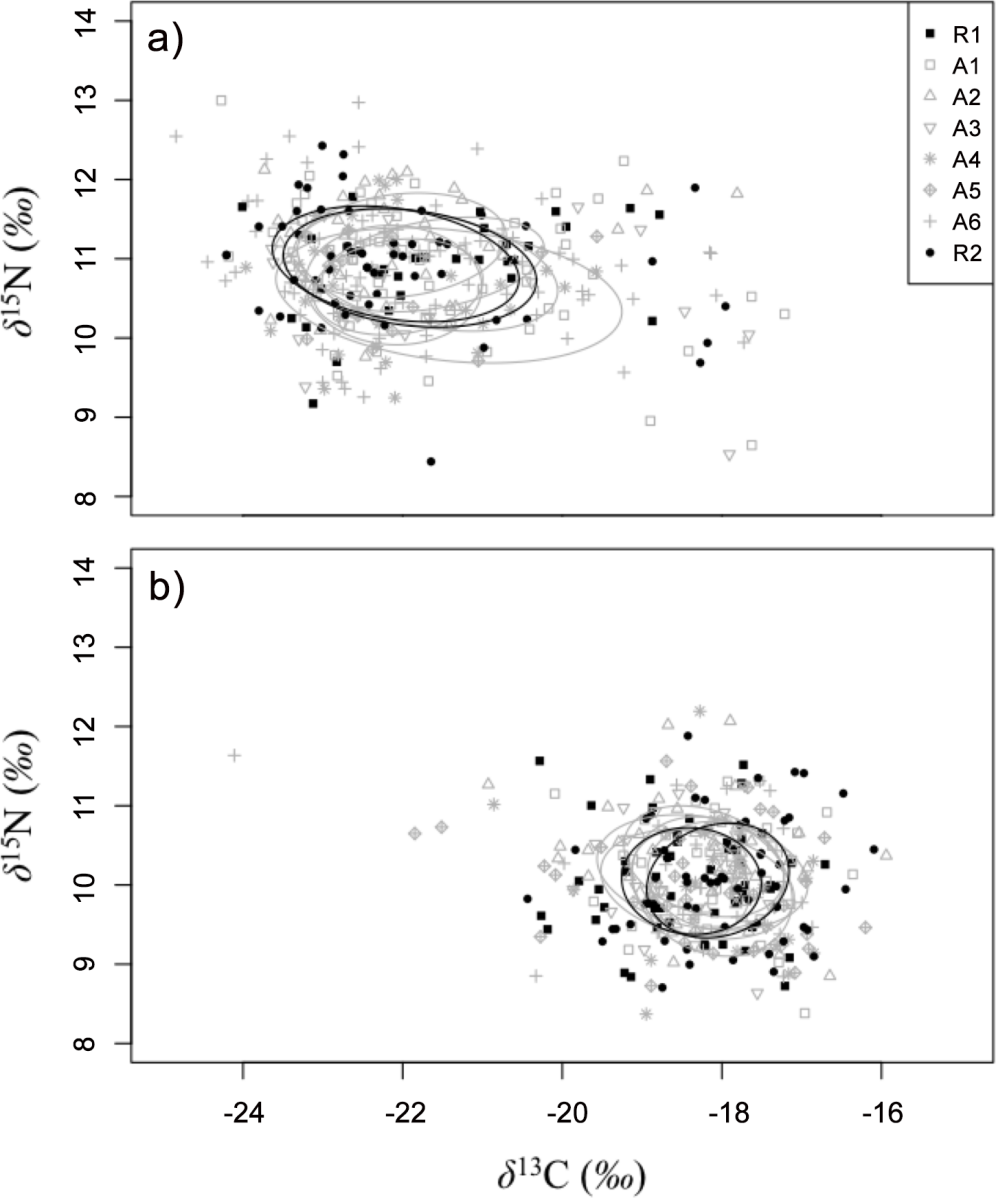
Bayesian standard ellipses describing isotopic niches of: (a) lake whitefish and (b) round whitefish collected in the fall of 2010 and 2011 from 8 sites in the Douglas Point area of Lake Huron. Fish were collected from two reference sites (black ellipses) and 6 potentially affected sites (grey ellipses). Isotopic values (δ^13^C and δ^15^N) for reference sites (closed symbols) and affected sites (open symbols) are derived from muscle tissue and shown in units of Per Mil (*‰)*. δ^13^C values are corrected for lipid content.

**Fig 3:**
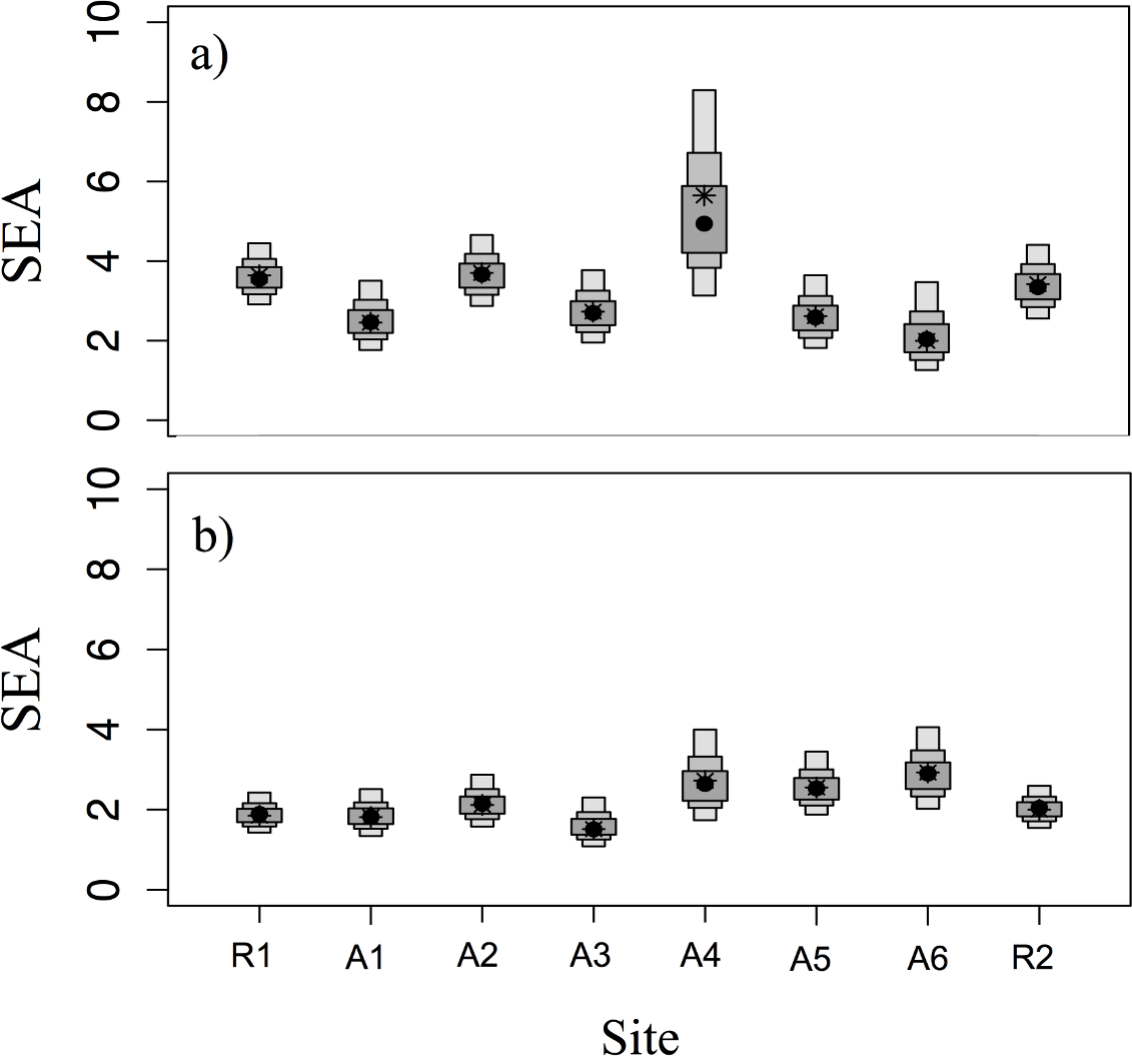
Density plot of Standard Ellipse Areas (SEA) for: (a) lake whitefish and (b) round whitefish collected from two reference sites (R1, R2) and 6 potentially affected sites (A1-A6) adjacent to Bruce Power on Lake Huron. SEA is the area (*‰*^2^) encompassed by standard ellipses (SE) and is a measure of isotopic niche size. The larger the SEA the more diverse the resource use is by fish. Boxes represent the 50%, 75% and 95% credible intervals of Bayesian estimates of SEA (10,000 replications), while black dots represent the mode SEA, and asterisks (*) represent SEA corrected for sample size (SEAc).

Mean *δ* ^13^C and *δ* ^15^N values were similar for fish of each species collected in reference sites to those in affected sites, but varied between species (Table 3). Correspondingly, the isotopic niches based on standard ellipses created using SIBER were largely overlapping within species (Fig 2). Average niche overlap between fish from affected sites and R1 was 82.0% ± 13.7% for lake whitefish and 72.9% ± 14.3% for round whitefish. Similarly, average niche overlap between R2 and affected areas was 77.0% ± 14.1% for lake whitefish and 75.7% ± 11.3% for round whitefish (Table 3). Thus, fish from reference and affected areas were using largely similar resources based on stable isotope values. Niche size (SEA_*C*_) was generally similar between reference and affected sites for lake whitefish, with the exception of site A4, which was substantially larger than all other sampling locations with high probability (Table 3 and Fig 3). For round whitefish, sites A4, A5 and A6 had larger SEA_*C*_ values than both reference sites and the remaining affected sites, also with high probability (Table 3 and Fig 3). Sites with larger niches corresponded with sites that had a smaller niche overlap with reference sites (Table 3).

**Table 3:** Isotopic niche data based on δ^13^C and δ^15^N values derived from adult lake and round whitefish muscle tissue. Fish were collected from two references sites (R1, R2), and 6 potentially affected sites (A1-A6) in the fall of 2010 and 2011. Standard Ellipse Area corrected for small sample size (SEA_*C*_) is a measure of niche size and resource use diversity. The percentage of isotopic niche overlap with reference sites, implying resource use similarity, is calculated for each site based on Bayesian standard ellipse overlap. The percent probability that niche size at each potentially affected site (SEA_A_) is larger than each reference site (SEA_R_) is calculated according to 10,000 Bayesian estimations of SEA. δ^13^C values are corrected for lipid.

Species & Site	*δ*^13^C	*δ*^15^N	C/N	SEA_*C*_	Niche overlap (%)		Prob. SEA_A_ > SEA_R_ (%)	
	Mean ± SD (‰)	Mean ± SD (‰)	Mean ± SD	(‰^2^)	R1	R2	R1	R2
Lake Whitefish								
R1	−21.9 ± 1.6	+10.9 ± 0.7	4.3 ± 0.6	3.64	100.0	88.8	-	-
A1	−21.6 ± 1.4	+10.9 ± 0.6	4.6 ± 0.7	2.45	90.3	84.6	5.0	10.3
A2	−21.7 ± 1.6	+10.9 ± 0.8	4.3 ± 0.5	3.70	90.4	80.6	55.1	66.9
A3	−22.0 ± 1.3	+11.2 ± 0.7	4.5 ± 0.7	2.73	84.0	82.6	8.9	17.4
A4	−21.4 ± 2.1	+10.5 ± 0.8	4.7 ± 0.7	5.65	54.6	48.6	94.1	95.4
A5	−22.1 ± 1.1	+10.7 ± 0.7	4.8 ± 0.9	2.61	84.1	80.5	6.3	12.6
A6	−22.1 ± 1.0	+10.6 ± 0.6	4.5 ± 0.7	1.99	88.6	85.2	3.8	6.6
R2	−22.1 ± 1.5	+10.9 ± 0.7	4.5 ± 0.7	3.42	94.5	100.0	-	-
Round Whitefish								
R1	−18.4 ± 0.9	+10.0 ± 0.7	4.0 ± 0.6	1.85	100.0	78.9	-	-
A1	−18.1 ± 0.8	+10.1 ± 0.7	4.1 ± 0.3	1.82	77.0	94.4	48.3	48.3
A2	−18.2 ± 1.0	+10.3 ± 0.7	4.0 ± 0.2	2.12	66.0	72.5	74.5	74.5
A3	−18.4 ± 0.7	+10.0 ± 0.7	4.1 ± 0.2	1.51	99.4	84.0	24.6	24.6
A4	−18.1 ± 1.0	+10.0 ± 0.9	3.8 ± 0.8	2.72	64.0	70.2	93.1	93.1
A5	−18.3 ± 1.2	+10.0 ± 0.7	3.8 ± 0.9	2.55	71.5	68.7	93.4	93.4
A6	−18.2 ± 1.3	+10.2 ± 0.7	3.7 ± 0.9	2.93	59.7	64.5	97.7	97.7
R2	−18.1 ± 0.9	+10.1 ± 0.7	3.7 ± 0.8	2.00	73.0	100.0	-	-

### Genetic Analysis

We genotyped 208 lake whitefish at 20 microsatellite loci and 327 round whitefish at 11 loci (Tables 2 and S2). For both species quality checks with MICRO-CHECKER did not indicate large allele dropout or scoring errors, although it did reveal that one of the lake whitefish loci (Cocl Lav45-tetra), showed significant null allele frequencies. Also, Cocl Lav1 was monomorphic so both of these loci were excluded from further analyses. None of the loci used to genotype round whitefish showed significant null allele frequencies. Tests using GENEPOP indicated that there were significant deviations from HWE in 7 (BWF2, Cocl Lav6-di, Cocl Lav20, Cocl Lav43, Cocl Lav44, Cocl Lav47 and Cocl Lav68) and 3 (Prwi27, Prwi60 and Prwi56) of the microsatellite loci for lake and round whitefish, respectively, following tests for multiple corrections. Thus after removing loci with significant nulls, monomorphic loci, and loci out of HWE, 11 and 8 loci were retained for lake and round whitefish, respectively.

When comparing the reference versus affected sites with all samples pooled, F_ST_ values were small and not significantly different from 0 (lake whitefish F_ST_ = 0.001, P = 0.252; round whitefish F_ST_ = 0.001, P = 0.070). The outcome was similar when comparing among sampling years (lake whitefish F_ST_ = 0.000, P = 0.481; round whitefish F_ST_ = 0.001, P = 0.298). Pairwise comparisons among zones also produced very small F_ST_ values not different from 0, with an average of -0.0015 for both lake and round whitefish (Tables 4 and 5).

**Table 4:** Pairwise estimates of F_ST_ among lake whitefish sampled from 8 different zones surrounding Bruce Power determined by pooling samples from 2010 and 2011. The F_ST_ value is found below the diagonal with the corresponding P-value above as determined by GENODIVE. These analyses only included 11 loci that were in Hardy-Weinberg equilibrium.

	R1	A1	A2	A3	A4	A5	A6	R2
R1	-	0.893	0.747	0.340	0.969	0.422	0.254	0.412
A1	-0.005	-	0.295	0.709	0.931	0.859	0.663	0.147
A2	-0.003	0.002	-	0.733	0.977	0.591	0.215	0.774
A3	0.001	-0.003	-0.003	-	0.832	0.336	0.185	0.104
A4	-0.010	-0.009	-0.010	-0.006	-	0.957	0.790	0.870
A5	0.000	-0.004	-0.001	0.001	-0.009	-	0.492	0.365
A6	0.004	-0.003	0.004	0.005	-0.007	-0.001	-	0.047
R2	0.001	0.004	-0.003	0.005	-0.006	0.001	0.012	-

**Table 5:** Pairwise estimates of F_ST_ among round whitefish sampled from 8 different sites surrounding Bruce Power determined by pooling samples from 2010 and 2011. The F_ST_ value is found below the diagonal with the corresponding P-value above as determined by GENODIVE. The analysis only included 8 loci that were in Hardy-Weinberg equilibrium.

	R1	A1	A2	A3	A4	A5	A6	R2
R1	-	0.134	0.072	0.376	0.650	0.054	0.423	0.242
A1	0.003	-	0.072	0.511	0.927	0.344	0.954	0.184
A2	0.003	0.005	-	0.926	0.927	0.625	0.278	0.544
A3	0.001	-0.001	-0.005	-	0.992	0.860	0.799	0.676
A4	-0.002	-0.006	-0.005	-0.010	-	0.954	0.838	0.966
A5	0.004	0.001	-0.001	-0.004	-0.006	-	0.819	0.334
A6	0.001	-0.005	0.002	-0.003	-0.004	-0.003	-	0.921
R2	0.001	0.002	-0.001	-0.002	-0.006	0.001	-0.003	-

**Fig 4:**
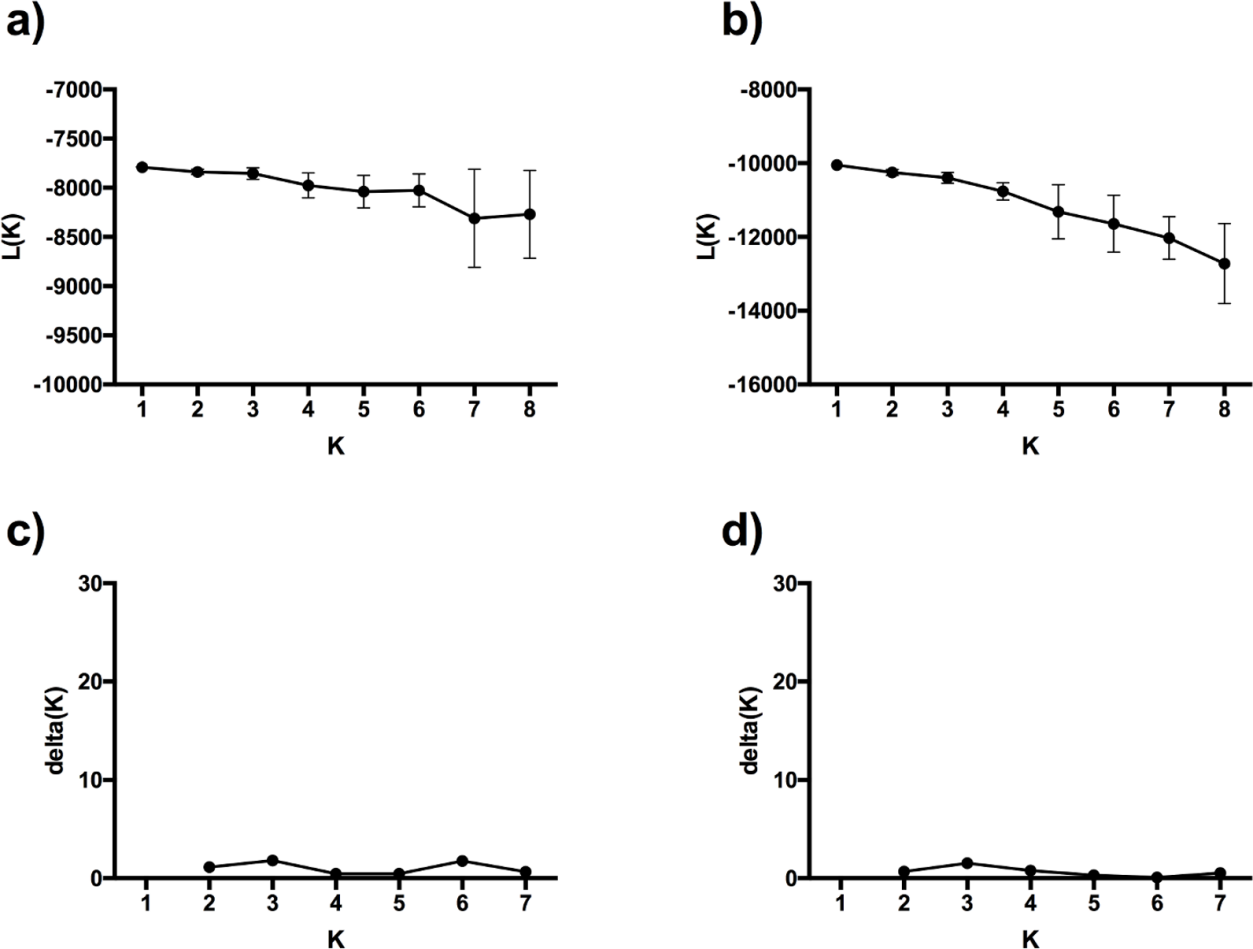
Probability output for the potential number of clusters (K) ranging from 1–8 with the program STRUCTURE for lake and round whitefish caught in the Douglas Point area. The procedure described in Pritchard et al. (2000) for: (a) lake whitefish and (b) round whitefish. Second order statistics from Evanno et al. (2005) for: (c) lake whitefish and (d) round whitefish.

Using the program STRUCTURE we evaluated models with the number of genetic clusters ranging from 1–8. The overall posterior probability estimates indicated that K = 1 had the highest probability for both species (Fig 4A and 4B). Further, there were no large peaks present when using the second-order statistics developed by Evanno *et al*. [88]. As this method is unable to evaluate a model of full panmixia, the lack of peaks implies the presence of one genetic cluster within the data (Fig 4C and 4D).

**Fig 5:**
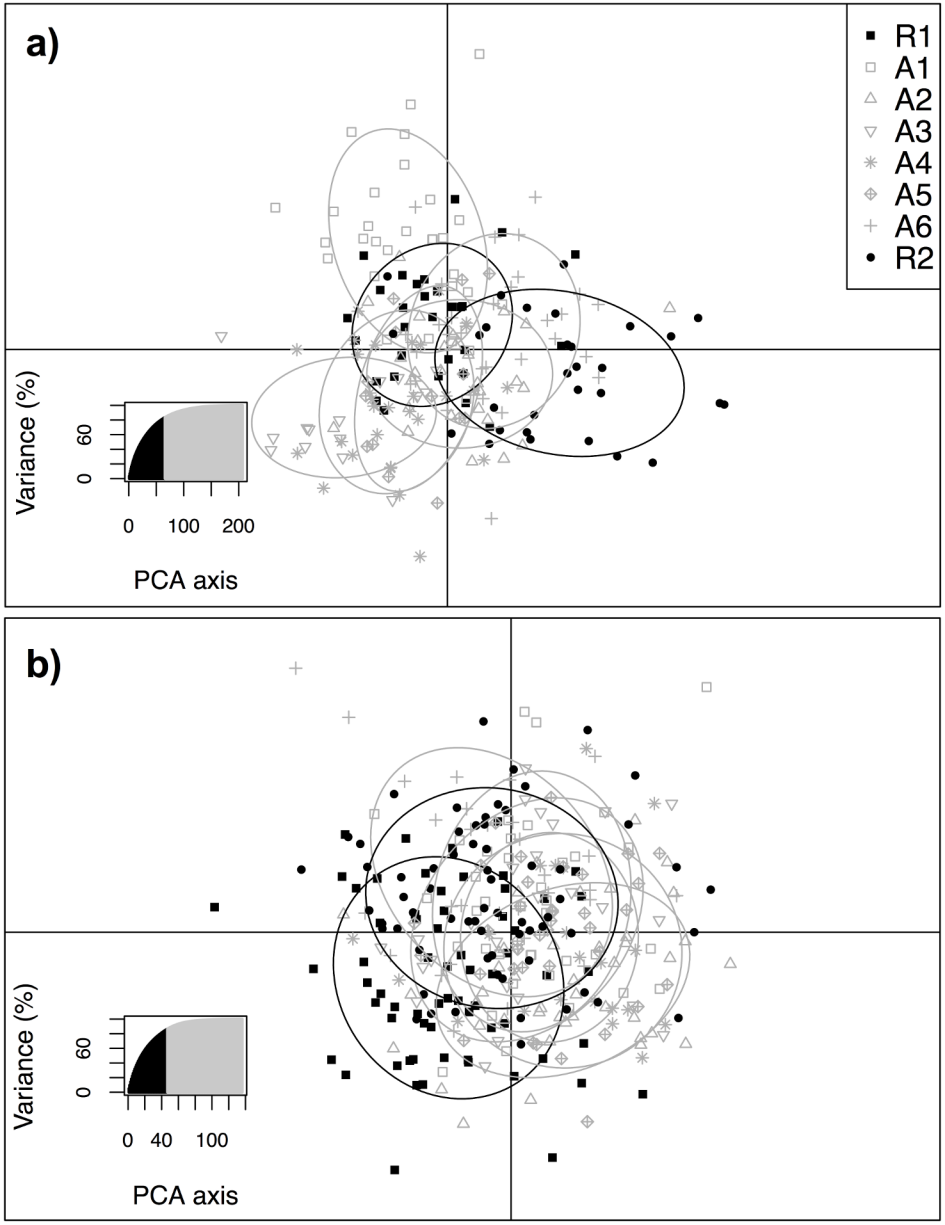
Discriminant analysis of principle components of: (a) lake whitefish and (b) round whitefish from 8 zones near Bruce Power on Lake Huron. Whitefish were collected from reference sites (black ellipses) and potentially affected sites (grey ellipses). Overlapping ellipses indicate no genetic differentiation.

The DAPC analysis was run with all loci and contained 83.3% and 86.0% of the variance within the data for lake and round whitefish, respectively. For lake and round whitefish the first two eigenvalues, the horizontal and vertical axes, were large, representing 47.3% and 42.3% of the variation, respectively, indicating that most of the between group variation was captured in the analysis. Overall, the DAPC analysis revealed overlapping, non-differentiated groupings by zone, further supporting the presence of one population for both lake and round whitefish (Fig 5).

## Discussion

Whitefish of the two species investigated were not ecologically or genetically distinct in areas affected by industrial thermal emissions compared to immediately adjacent reference zones. Thus, fish in both the affected and external reference areas appear to be part of a larger population extending outside of the study area. Our findings suggest that thermal emissions from nuclear power generation are unlikely to be directly affecting a local population of either species that is an evolutionarily significant unit [90–95]. However, this finding does not negate the need for management considerations and monitoring in our study area. Elevated temperatures during development reduce survival of embryos and fry [3,7,9,51], potentially reducing the productivity of fish spawning in areas affected by thermal emissions. Previous studies have also found that thermal pollution can change fish behavior [5,6,11,96,97], potentially influencing fish recruitment. Future studies will need to determine how important the spawning habitat is in our study area and estimate how potential thermal effects may affect recruitment.

Based on stable isotopes, the fish captured in affected and reference areas came from similarly diverse food webs. Both lake and round whitefish showed extensive resource use heterogeneity, but mean *δ* ^13^C and *δ* ^15^N values and niche-overlap analyses indicated that within species they used similar resources across reference and affected zones. Large lakes can have broad geographic and depth-based variation in baseline stable isotopes values (e.g., [63,98–100], which provides the opportunity to detect ecological (isotopic) population structure among groups based on location and habitat use. Thus, the adult fish we analyzed from both species likely spent time in a variety of areas in Lake Huron with different isotopic baselines during the summer prior to moving into near-shore areas around Douglas Point in the fall (several months for muscle turnover time; [74,101]). Consequently, spawning fish in our study area are mixed aggregations from several food webs rather than local stocks, a phenomenon also observed in other parts of Lake Huron [58].

Niche size was larger for both whitefish species in the areas expected to be most influenced by thermal emissions. This effect was most pronounced for lake whitefish, which had an SEA_*C*_ value 1.5- to 2.8-fold higher in Area 4, immediately adjacent to the larger thermal outflow, than any other zone. Similarly, round whitefish SEA_*C*_ values were 1.3- to 1.5-fold higher in Areas 4 to 6 than in the other areas studied. Comparable differences in SEA_c_ have been documented between aquatic consumers of different feeding guilds [102] and between sympatric fish species [103]. Thus, the differences we observed in SEAc between fish in some affected vs. reference areas are likely biologically relevant differences in resource use diversity. Based on the rationale provided above, levels of fish mixing in the affected areas were higher than in the reference zones. Importantly, this suggests that fish in the most affected areas relied on a more diverse array of diets and habitats, rather than using distinct resources, which has been documented as 0% isotopic niche overlap [104]. In contrast our niche overlap between reference and affected areas ranged from 48.6 to 94.4%. We propose two potential explanations for these findings that are not mutually exclusive: (1) habitat in the thermally affected areas is desirable and attracts more mixing of fish from multiple feeding locations (see [5,105]); or (2) the fish captured in affected zones are not actually spawning there, and our samples contain a more diverse collection of individuals that are simply moving through those areas (whitefish make near shore movements throughout the fall; [58]). Our data do not permit us to distinguish between these explanations, but reinforce the need to understand the importance of our study area for actual spawning activities as identified above.

There was no genetic differentiation between affected and reference zones for either lake or round whitefish, which were both part of single populations across our study area. This finding was not unexpected for lake whitefish, which have significant population structure throughout the Great Lakes system [48,106], and in Lake Huron, but over much larger geographic scales than we studied [14,19,57,59]. In particular, Stott *et al*. [19] showed no differentiation between fish from Douglas Point and the Fishing Islands more than 70 km to the north. However, in that study no sites were sampled between Douglas Point and the Fishing Islands, and no sampling was conducted to the south. Further, the genotyping only consisted of seven microsatellite loci, which may result in reduced power to detect differentiation between sites [107–111]. Interestingly, although there were no distinct genetic clusters present in our DAPC analysis for lake whitefish, comparing the ellipses for the northern most sites, R1, A1 and A2, to the southern reference site, R2, suggests that spawning aggregations farther south are less similar. Additional research will be required to determine the full extent of lake whitefish genetic structuring within Lake Huron with finer resolution to determine how those sampled at Douglas Point fit into the overall structure within the lake.

Round whitefish presented essentially a complete unknown in terms of *a priori* expectations about population structure. Lake whitefish have been much more intensively studied because of their commercial value (e.g., [48,106,112–117]), but interest in genetic studies of round whitefish has only recently emerged (e.g., [81,118]). Round whitefish are ecologically distinct from lake whitefish [44,51,113,119,120] and little is known about their movements or spawning site fidelity. Similar to lake whitefish, round whitefish did not show any genetic population subdivision over our study area. DAPC analyses, our most powerful tool for examining structure, produced ellipses that were completely overlapping and smaller and more focused on the center of the axes than those for lake whitefish. Thus, round whitefish showed essentially no differentiation over the area sampled, which is emphasized by the DAPC analysis where the variance between groups is maximized. Given the limited geographic scope of our study, we can conclude only that there is no genetic distinction between fish captured inside and outside of the thermally affected area. However, round whitefish populations have drastically declined in Lake Huron in recent years and concern for this species is growing [49]. To ensure effective management of this species a large-scale genetic study is required to determine the population structure of round whitefish within their range.

Our study ultimately suggests that concerns over the impacts of thermal effluent should be more focused on potential changes to productivity and recruitment rather than conservation of discrete populations. Both ecological and genetic data indicate that adult fish inside and immediately outside of thermally affected areas are part of larger genetic populations that use similarly diverse resources. Thus, adverse effects of thermal emissions, if any, would be limited to some fraction of a larger genetic and ecological group for both study species. Large-scale commercial harvest of lake whitefish occurs in the Fishing Islands area north of our study location [58]. Our data and those of Stott *et al*. [19] suggest that fish from Douglas Point are part of a larger population that includes the Fishing Islands. Previous work has shown that the habitat within the vicinity of the power plant attracts ripe and mature whitefish [67], so potential reductions in productivity of spawning fish in the Douglas Point area may be a fisheries management concern if local spawning produces a significant number of recruits for harvest. This information is currently unavailable given limited knowledge of spawning activity in our study area. However, recent in situ work has shown only small changes in temperature with little predicted change to developmental timing of lake whitefish eggs [65]. Thus, thermal impacts on lake whitefish productivity are likely to be minor in comparison to the commercial harvest of over 1 million kilograms within the management unit that includes Douglas Point [56]. In general, the effects of industrial pollution can vary depending on the system, so careful attention to context is required (e.g., [1,3,6–8,11,51,121–123]).

## Supporting Information

S1 Table Details for 16 polymorphic microsatellite loci developed for lake and round whitefish specifically for this study. The size indicates the range of observed alleles in base pairs and includes the length of the CAG tag; number of individuals genotyped is *N*; *k* is number of alleles observed; H_o_ and H_e_ are observed and expected heterozygosity, respectively; PI is the probability of identity for each locus, and TD refers to the touchdown protocol used for PCR (see text).
(DOCX)

S2 Table Summary data for the 31 microsatellite loci used to genotype lake and round whitefish for this study. The repeat size indicates the length of the repeat within each locus; N is the number of individuals genotyped; *k* refers to the number of alleles observed; H_O_ and H_E_ are observed and expected heterozygosities, and PIC is the polymorphic information content.
(DOCX)
