# Supplementary material for: Fine-Scale Ecological and Genetic Population Structure of Two Whitefish (Coregoninae) Species in the Vicinity of Industrial Thermal Emissions
Source: PLoS One. 2016 Jan 25;11(1):e0146656. doi: 10.1371/journal.pone.0146656 (PMC4726566; doi:10.1371/journal.pone.0146656)
Supplement: S1 Table — The size indicates the range of observed alleles in base pairs and includes the length of the CAG tag; number of individuals genotyped is N; k is number of alleles observed; Ho and He are observed and expected heterozygosity, respectively; PI is the probability of identity for each locus, and TD refers to the touchdown protocol used for PCR (see text). (DOCX) [file pone.0146656.s001.docx]

**Table S1.** Details for 16 polymorphic microsatellite loci developed for lake and round whitefish specifically for this study*.* The size indicates the range of observed alleles in base pairs and includes the length of the CAG tag; number of individuals genotyped is *N*; *k* is number of alleles observed; H_o_ and H_e_ are observed and expected heterozygosity, respectively; PI is the probability of identity for each locus, and TD refers to the touchdown protocol used for PCR (see text).

| Locus | Primer Sequence 5’ -> 3’ | Repeat Motif | Size (bp) | N | *k* | H_o_ | H_e_ | PI | TD |
| --- | --- | --- | --- | --- | --- | --- | --- | --- | --- |
| *Lake Whitefish* |  |  |  |  |  |  |  |  |  |
| Cocl Lav6 | F: *AGGGACTCTGCTTTATTTCTCC  R: AGAACGAGTGAATGAGGAAGG | AAAG | 183-243 | 24 | 13 | 0.958 | 0.898 | 0.019 | TD65 |
| Cocl Lav12 | F: *CCGTTCTGGACGACTTGAGC  R: CAGATATTAGACCGAGTTTGTGTTGC | AAAG | 196-274 | 24 | 15 | 0.708 | 0.815 | 0.056 | TD65 |
| Cocl Lav18 | F: *CCACTACAGATAAACACAGACAGGC  R: CTGCAGTACAGCTCCAGCC | TCTG | 188-220 | 24 | 9 | 0.792 | 0.850 | 0.040 | TD65 |
| Cocl Lav20 | F: *TGATACAGCATGTTGTCAGATGG  R: GAAACTAACACACACCAGAATGAACC | TCTG | 316-348 | 24 | 10 | 0.917 | 0.843 | 0.043 | TD65 |
| Cocl Lav33 | F: *GAGCAGGAGAGCAACACAACC  R: TCAATGCTCAGCTCTCAAAGG | AGTG | 176-232 | 24 | 15 | 0.958 | 0.914 | 0.014 | TD65 |
| Cocl Lav34 | F: *TGTGGGACATGAAGAGGAGG  R: TCACTTGTACATACACTGAAACAAATCC | AGTG | 212-232 | 24 | 6 | 0.750 | 0.708 | 0.130 | TD65 |
| Cocl Lav43 | F: *GGTTTCTCCAGTGAGATCAAAGC  R: TTAACCTGGCTTTGTGGTGC | TCTG | 205-277 | 23 | 14 | 0.783 | 0.904 | 0.017 | TD65 |
| Cocl Lav44 | F: *TTCTGCTCATGAAACACGGG  R: TTCACAGTATTATTCCAACCTCATCC | TCTG | 128-180 | 23 | 11 | 0.783 | 0.860 | 0.034 | TD65 |
| Cocl Lav45 | F: *TGCGAAACGACTCCCACC  R: TTCTGACACATGACTTACAGTATTCCC | TCTG | 168-244 | 23 | 16 | 0.913 | 0.909 | 0.015 | TD65 |
| Cocl Lav47 | F: *TGCCTACCTCTCGTGTGCC  R: TTGTTTCTATCAATGCACCATCC | ATGG | 318-356 | 23 | 10 | 0.783 | 0.821 | 0.053 | TD65 |
| Cocl Lav48 | F: *TGCCTGTCTGCATCTGCC  R: TTTCCACTGCGGATTTACCC | TCTG | 322-398 | 19 | 14 | 0.737 | 0.900 | 0.018 | TD65 |
| *Round Whitefish* |  |  |  |  |  |  |  |  |  |
| Prwi55 | F: *TCATTATTACTGACACAGATAGACGG  R: CAGATTAATCAGATACTGCTAGCCC | TCTG | 138-162 | 24 | 6 | 0.833 | 0.747 | 0.105 | TD65 |
| Prwi56 | F: *GGCTCTGGCTGCTTTCTAGC  R: CATGAACCCTCTGCGAACC | AAAG | 394-413 | 22 | 6 | 0.455 | 0.546 | 0.25 | TD65 |
| Prwi60 | F: *ACTTCTATACAGTCATCATCTGCCC  R: GCAATTTCATAAATGCCTGCC | TGCC | 163-199 | 18 | 7 | 0.778 | 0.773 | 0.11 | TD65 |
| Prwi65 | F: *TCATTAACCTACAGCTATTACAGAGGC  R: GGTCTGTAGCTGTCGGGC | TCTG | 312-340 | 24 | 8 | 0.792 | 0.707 | 0.11 | TD65 |
| Prwi72 | F: *GGCTGACACAGTAAGAGGGC  R: TTGGTGTGATGCAATACAGTAGC | TCTG | 244-284 | 24 | 13 | 0.833 | 0.842 | 0.04 | TD65 |

* indicates CAG tag (5’- CAGTCGGGCGTCATCA -3’) label;
