# Supplementary material for: Fine-Scale Ecological and Genetic Population Structure of Two Whitefish (Coregoninae) Species in the Vicinity of Industrial Thermal Emissions
Source: PLoS One. 2016 Jan 25;11(1):e0146656. doi: 10.1371/journal.pone.0146656 (PMC4726566; doi:10.1371/journal.pone.0146656)
Supplement: S2 Table — The repeat size indicates the length of the repeat within each locus; N is the number of individuals genotyped; k refers to the number of alleles observed; HO and HE are observed and expected heterozygosities, and PIC is the polymorphic information content. (DOCX) [file pone.0146656.s002.docx]

**Table S2.** Summary data for the 31 microsatellite loci used to genotype lake and round whitefish for this study. The repeat size indicates the length of the repeat within each locus; N is the number of individuals genotyped; *k* refers to the number of alleles observed; H_O_ and H_E_ are observed and expected heterozygosities, and PIC is the polymorphic information content.

| Locus | Repeat Size | N | *k* | H_O_ | H_E_ | PIC |
| --- | --- | --- | --- | --- | --- | --- |
| *Lake Whitefish* |  |  |  |  |  |  |
| BWF1 | Di | 179 | 11 | 0.693 | 0.726 | 0.683 |
| BWF2 | Di | 177 | 10 | 0.571 | 0.649 | 0.609 |
| Cocl Lav1 | Di | 166 | 2 | 0.006 | 0.006 | 0.006 |
| Cocl Lav4 | Di | 178 | 4 | 0.629 | 0.654 | 0.579 |
| Cocl Lav6 | Di | 179 | 9 | 0.346 | 0.479 | 0.462 |
| Cocl Lav6 | Tetra | 205 | 18 | 0.839 | 0.915 | 0.907 |
| Cocl Lav12 | Tetra | 204 | 20 | 0.824 | 0.835 | 0.817 |
| Cocl Lav18 | Tetra | 205 | 10 | 0.878 | 0.840 | 0.818 |
| Cocl Lav19 | Di | 179 | 11 | 0.642 | 0.643 | 0.603 |
| Cocl Lav20 | Tetra | 205 | 17 | 0.829 | 0.906 | 0.896 |
| Cocl Lav27 | Di | 176 | 4 | 0.080 | 0.088 | 0.085 |
| Cocl Lav33 | Tetra | 204 | 18 | 0.912 | 0.911 | 0.902 |
| Cocl Lav34 | Tetra | 195 | 10 | 0.718 | 0.770 | 0.739 |
| Cocl Lav43 | Tetra | 204 | 35 | 0.750 | 0.941 | 0.936 |
| Cocl Lav44 | Tetra | 206 | 32 | 0.840 | 0.921 | 0.914 |
| Cocl Lav45 | Di | 163 | 8 | 0.613 | 0.699 | 0.646 |
| Cocl Lav45 | Tetra | 180 | 17 | 0.456 | 0.903 | 0.891 |
| Cocl Lav47 | Tetra | 205 | 19 | 0.761 | 0.923 | 0.915 |
| Cocl Lav48 | Tetra | 205 | 30 | 0.922 | 0.939 | 0.933 |
| Cocl Lav68 | Di | 162 | 5 | 0.358 | 0.361 | 0.322 |
| *Round Whitefish* |  |  |  |  |  |  |
| Prwi6 | Tetra | 324 | 11 | 0.639 | 0.636 | 0.590 |
| Prwi15 | Tetra | 316 | 22 | 0.829 | 0.861 | 0.844 |
| Prwi24 | Tetra | 318 | 33 | 0.868 | 0.911 | 0.903 |
| Prwi25 | Tetra | 324 | 9 | 0.614 | 0.638 | 0.587 |
| Prwi27 | Tetra | 324 | 12 | 0.775 | 0.8080 | 0.779 |
| Prwi28 | Tetra | 322 | 16 | 0.904 | 0.901 | 0.891 |
| Prwi55 | Tetra | 324 | 14 | 0.759 | 0.769 | 0.735 |
| Prwi72 | Tetra | 323 | 15 | 0.854 | 0.878 | 0.865 |
| Prwi60 | Tetra | 308 | 11 | 0.698 | 0.781 | 0.748 |
| Prwi56 | Tetra | 317 | 11 | 0.530 | 0.618 | 0.573 |
| Prwi65 | Tetra | 318 | 10 | 0.701 | 0.713 | 0.688 |
